# Construction of S-Scheme 2D/2D Crystalline Carbon Nitride/BiOIO_3_ van der Waals Heterojunction for Boosted Photocatalytic Degradation of Antibiotics

**DOI:** 10.3390/molecules28135098

**Published:** 2023-06-29

**Authors:** Xiangyuan Kong, Longwen Cao, Yuxing Shi, Zhouze Chen, Weilong Shi, Xin Du

**Affiliations:** 1 School of Physical Science and Engineering, Beijing Jiaotong University, Beijing 100091, China; 2 School of Material Science and Engineering, Jiangsu University of Science and Technology, Zhenjiang 212003, Chinaczz990707@163.com (Z.C.); 3 College of Chemistry, Zhengzhou University, Zhengzhou 450001, China

**Keywords:** S-scheme, 2D/2D, crystalline carbon nitride, BiOIO_3_, van der Waals heterojunction

## Abstract

Utilization of semiconductor photocatalyst materials to degrade pollutants for addressing environmental pollution problems has become a research focus in recent years. In this work, a 2D/2D S-scheme crystalline carbon nitride (CCN)/BiOIO_3_ (BOI) van der Waals heterojunction was successfully constructed for effectively enhancing the degradation efficiency of antibiotic contaminant. The as-synthesized optimal CCN/BOI-3 sample exhibited the highest efficiency of 80% for the photo-degradation of tetracycline (TC, 20 mg/L) after 120 min visible light irradiation, which was significantly higher than that of pure CCN and BOI. The significant improvement in photocatalytic performance is mainly attributed to two aspects: (i) the 2D/2D van der Waals heterojunction can accelerate interface carriers’ separation and transfer and afford sufficient active sites; (ii) the S-scheme heterojunction elevated the redox capacity of CCN/BOI, thus providing a driving force for the degradation reaction. The degradation pathways of TC for the CCN/BOI composite were investigated in detail by liquid chromatography-mass spectrometry (LC-MS) analysis. This work provides a design idea for the development of efficient photocatalysts based on the 2D/2D S-scheme van der Waals heterojunctions.

## 1. Introduction

Over the past few decades, antibiotics have been used in a wide variety of fields and have become an essential part of people’s lives. However, overuse and mishandling of antibiotics makes it easy for them to accumulate in the environment and pose a health risk to humans and other organisms [1]. There is thereby an urgent need to remove antibiotics from the environment, but it is still a significant challenge. In recent years, researchers have tried to use conventional physical adsorption, biological filtration, chemical treatment, and other methods to eliminate antibiotics [2,3,4,5]. Unfortunately, these methods are difficult to apply in practice due to process complexity, the cost of the facilities, and potential secondary contamination [6,7,8].

Recently, semiconductor photocatalysis has attracted a great deal of attention due to its ability to harness solar energy efficiently and its economic applicability. Among the various photocatalysts, carbon nitride (CN) stands out due to its non-toxicity, stability, excellent optical properties, and suitable electronic structure [9,10]. Nevertheless, some inherent characteristics of CN, such as easy recombination of photo-generated carriers, low specific surface area, and its few active sites, greatly limit the application of CN in the photocatalytic field [11,12]. To overcome the shortcomings of CN, researchers have developed numerous strategies for its use, such as designing morphological structures [13,14,15,16,17], building heterojunctions [18,19,20,21], and improving crystallinity [22,23,24]. Among these modification methods, it was found that increasing crystallinity to form crystalline CN (CCN) could remove the high-density defects and terminal amino groups in CN, thus effectively inhibiting the recombination of photogenerated electron-hole pairs and enhancing the charge carrier migration rate of CN [25,26]. Furthermore, hybridizing two-dimensional (2D) CN with other semiconductors to construct a 2D/2D van der Waals (VDW) heterojunctions is also a promising strategy for enhancing the photocatalytic activity of CN. Compared to normal heterojunctions, the 2D/2D VDW heterojunctions possess strong interactions and large interface areas due to the unique morphology of materials, which provides channels for the transportation of charge carriers and facilitates carrier transfer rate [27,28]. Moreover, the transfer path of photo-generated electrons in the heterojunction also plays an important role in the effectiveness of the fabricated photocatalyst [29,30]. Notably, step-scheme (S-scheme) heterojunctions raised from Yu’s group possesses various unique advantages, which not only promote photo-induced charge separation but also enable the photocatalyst to keep stronger reduction and oxidation capabilities [31,32,33]. In S-scheme heterojunctions, the *e^−^* and *h^+^* spatial separations are located on the conduction band (CB) and valence band (VB), respectively. Accordingly, *e^−^* tends to remain at a more negative potential for reduction, while *h^+^* tends to remain at a more positive potential for oxidation, resulting in a stronger redox ability while effectively inhibiting charge recombination [34,35,36,37,38]. Consequently, the construction of a 2D/2D S-scheme CCN-based VDW heterojunction is necessary for the development and utilization of the enhanced treatment of degraded wastewater with various antibiotics.

Bismuth-based semiconductors have recently become a hot topic in the field of photocatalysts [37]. Bismuth oxiodate (BiOIO_3_), a new member of the bismuth-based semiconductor family, exhibits excellent photocatalytic activity due to its favorable physicochemical properties and unique asymmetric sheet structure [39,40]. Furthermore, the two isolated cation pairs (Bi^3+^ and I^5+^) of BiOIO_3_ and the structure exhibiting an Aurivillius-type (Bi_2_O_2_)^2+^ layer with an intercalated (IO_3_)^−^ anion can play a role in promoting photo-induced carrier migration and separation [41]. It is worth noting that BiOIO_3_ with a wide band gap (3.1 eV) is one of the most suitable candidates to meet the energy band alignment to construct heterojunctions with CN [42]. Consequently, inspired by the above considerations, the construction of S-scheme 2D-2D VDW heterojunctions between BiOIO_3_ and CCN could create an even more excellent activity.

In this work, an S-scheme 2D/2D CCN/BOI VDW heterojunction photocatalyst was successfully fabricated via a simple hydrothermal method, which exhibited excellent photocatalytic activity in degrading tetracycline antibiotics. Moreover, the as-synthesized materials achieved the best photocatalytic performance by adjusting the ratio of CCN to BOI, which is remarkably higher than that of pure CCN and BOI. Furthermore, the enhanced photocatalytic performance of CCN/BOI composites and the reaction mechanism of S-scheme 2D/2D VDW heterojunctions were investigated and discussed through a series of characterization analyses and theoretical calculations. 

## 2. Results and Discussion

**Figure 1:**
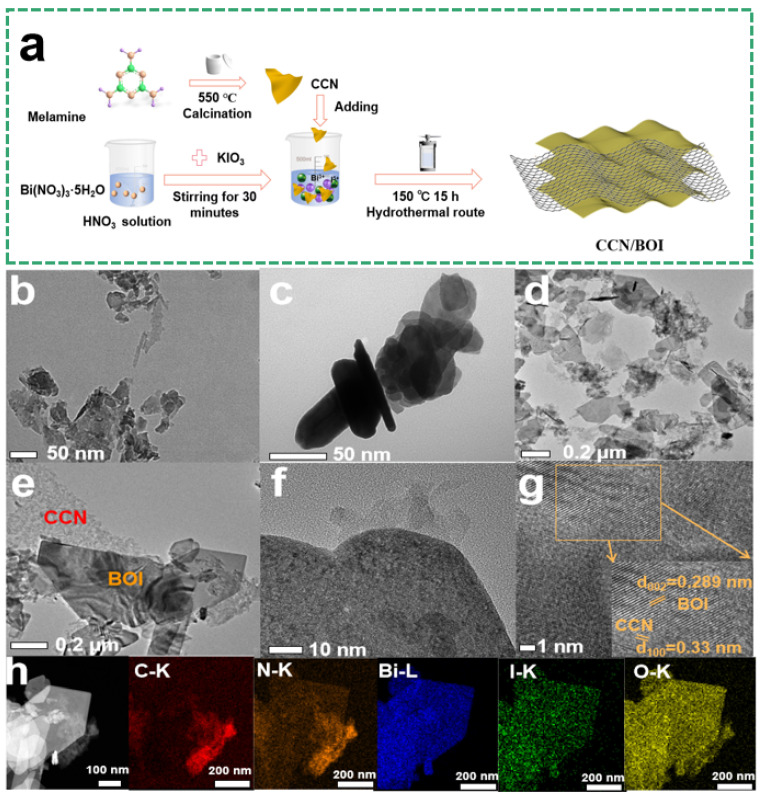
(**a**) Schematic diagram of the synthetic route over CCN/BOI heterojunction photocatalyst. TEM images of (**b**) CCN, (**c**) BOI, (**d**,**e**) CCN/BOI-3. (**f**,**g**) HRTEM images of CCN/BOI-3. (**h**) HAADF and elemental mapping images of CCN/BOI-3.

The diagram of the synthesis mechanism of 2D/2D CCN/BOI heterojunction is displayed in Figure 1a. Initially, the CCN nanosheets were synthesized by a one-step rapid polymerization strategy by direct calcination of melamine at 550 °C, without an early heating process, and also without the help of any additives or salt intercalation. Subsequently, during the hydrothermal reaction, the BOI nanosheets were fabricated by controlling the acidity of the solution, while the CCN nanosheets with a large H^+^ thermodynamic driving force were successfully coupled to the BOI nanosheets by the VDW driving force to form a 2D/2D CCN/BOI heterojunction. Furthermore, the morphological features and crystal structures of the as-synthesized samples were clearly characterized by transmission electron microscopy (TEM) and high-resolution TEM (HRTEM). As displayed in Figure 1b, the pure CCN exhibits an irregular layered nanosheet structure, while the bare BOI presents a thin nanosheet morphology (Figure 1c). Notably, the scanning electron microscopy (SEM, Figure S1a) image reveals that the overall morphology of the composite remains as a lamellar structure, and the energy dispersive X-ray spectrum (EDS, Figure S1b) demonstrates the presence of the corresponding C, N, Bi, O, and I elements in the material. Furthermore, the large area and tight contacts at the 2D/2D interface can facilitate the formation of heterojunctions (Figure 1d,e). Remarkably, in the HRTEM image (Figure 1f) of the CCN/BOI-3 composite, it can be observed that the CCN nanosheets are attached to the surface of the BOI nanosheets. Meanwhile, the two different lattice fringes can be noticed in the local magnification of the HRTEM image in Figure 1g, and the 0.289-nm and 0.33-nm lattice fringes belong to the (002) plane of BOI and the (002) plane of CCN, respectively [43,44]. Notably, the observed intimate interface between CCN and BOI implies that the final synthesized CCN/BOI compounds has been obtained by van der Waals heterojunctions. Furthermore, the high-angle ring dark-field (HAADF) and the corresponding elemental mapping images (Figure 1h) display that the elements of C, N, Bi, I, and O are uniformly distributed in the CCN/BOI-3 composite, which indicates that the CCN has been successfully coupled to the BOI.

**Figure 2:**
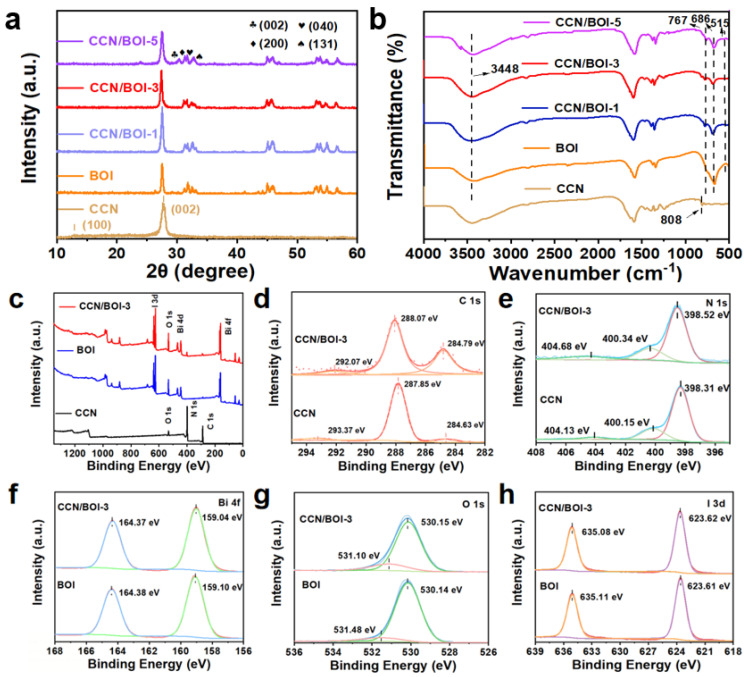
(**a**) XRD patterns and (**b**) FT-IR spectra of the as-synthesized CCN, BOI, and CCN/BOI heterojunctions. (**c**) XPS survey and high-resolution spectra of CCN, BOI, and CCN/BOI-3: (**d**) C 1s; (**e**) N 1s; (**f**) Bi 4f; (**g**) O 1s; (**h**) I 3d.

The composition, phases, and crystal structures of pure CCN, BOI, and CCN/BOI composites were determined by X-ray diffraction (XRD), and the results are displayed in Figure 2a. For bare CCN, the two different diffraction peaks located at 13.3° and 27.4° can be attributed to the (100) and (002) crystal planes of CN [45], which correspond to the in-plane stacking structure of the conjugated aromatic group and the in-plane structural stacking of the tris-triazine unit, respectively [46]. Compared with the BCN, the diffraction peak intensity of the CCN increased significantly, indicating an improved crystallinity (Figure S2). For pure BOI, the characteristic peaks at 27.36°, 31.11°, 31.61°, 32.46°, and 32.96° can be indexed to (121), (002), (200), (040), and (131) crystal planes of the orthorhombic BiOIO_3_ (JPCDS No. 26-2019), respectively [47]. Meanwhile, for the profile of the as-synthesized composites CCN/BOI, the diffraction peaks can be indexed to BOI and CCN, which indicates the successful coupling of CCN with BOI. Furthermore, the Fourier transform infrared spectroscopy (FT-IR) spectra of the as-synthesized samples are exhibited in Figure 2b. For pristine CCN, the peak at 808 cm^−1^ corresponds to the characteristic breathing pattern of the s-triazine unit, while the peak in the region around 3000 cm^−1^ is attributed to the stretching vibration of the N-H bond [48]. For pure BOI, the two peaks located at 686 and 767 cm^−1^ are ascribed to stretching vibrations of the I-O bond, and the peak at 515 cm^−1^ is associated with stretching vibrations of the Bi-O bond [49,50]. Furthermore, the broad peak at 3430 cm^−1^ is assigned to a bending vibration of the O-H group, indicating the presence of hydroxyl groups on the surface of BOI [51]. The characteristic peaks belonging to both CCN and BOI are observed in CCN/BOI composites, which indicates that the CCN and BOI are indeed coupled, in agreement with the XRD results. The elemental chemical states, compositions, and interactions between CCN and BOI were characterized by X-ray photoelectron spectroscopy (XPS) measurement. The full XPS survey spectra in Figure 2c confirm that C, N, Bi, O, and I elements all exist in the as-synthesized CCN/BOI-3 composite. As exhibited in Figure 2d, the strong peaks located at 284.63, 287.85, and 293.37 eV in CCN are assigned to the C-C single bond with sp^2^ hybridization, the sp^2^-hybrid C (N-C=N) coordination in the triazine ring, and π-excitation, respectively [52]. In Figure 2e, the N 1s spectrum of CCN exhibits three peaks at 398.31, 400.15, and 404.13 eV, which are ascribed to the sp^2^ nitrogen atoms (C-N=C), N-(C)_3_ groups, and N-H bonds, respectively [53,54]. The Bi 4f spectrum of BOI consists of two peaks at 159.10 and 164.38 eV, as seen in in Figure 2f, which correspond to Bi 4f_7/2_ and Bi 4f_5/2_, indicating that Bi exists in the state of Bi^3+^ [40]. In Figure 2g, the O 1s spectrum of BOI can be deconvoluted into two peaks at 530.14 and 531.48 eV, corresponding to the lattice oxygen I-O [7]. Furthermore, the I 3d spectrum of BOI exhibited two peaks at 623.61 and 635.11 eV (Figure 2h), belonging to I 3d_5/2_ and I 3d_3/2_, indicating that element I is present in the +5 valence state [55]. Notably, the shift of binding energy clearly demonstrates the transfer of photo-induced electrons from BOI to CCN, indicating the strong coupling between CCN and BOI with the formation of heterojunctions in the BOI/CCN composite.

**Figure 3:**
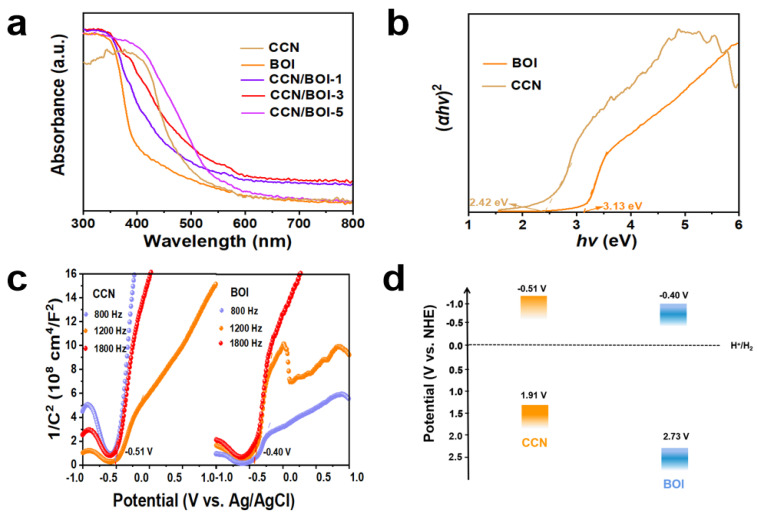
(**a**) Diffuse reflectance spectra of as-prepared samples. (**b**) Kubelka–Munk plots of CCN and BOI. (**c**) M–S plots and (**d**) the corresponding band structures of CCN and BOI.

The optical absorption properties of the as-synthesized samples were analyzed by UV-vis diffuse-reflectance measurement, and the results are exhibited in Figure 3a. It can be observed that the absorption peaks of pure CCN and BOI are located at 460 and 405 nm, respectively, which are mainly attributed to the intrinsic band gaps of CCN and BOI [56]. With the gradual increase of the introduced CCN content, the absorption edge of CCN/BOI-x composites appear to red-shift and the light absorption intensities have been significantly enhanced (digital photographs are given in Figure S3), which are mainly ascribed to the successful combination of CCN and BOI to promote the transfer of photogenerated carriers and substantially improve the light utilization efficiency. Moreover, the band gap energies of pure CCN and BOI can be calculated by the following equations: *αhv* = A(hv − E_g_)^n/2^, where α, *hv*, A, E_g_, and n are the absorption coefficient, photon energy, constant, band gap energy, and constant determined by the type of optical transition of the semiconductor material, respectively [57]. Based on the equation, the calculated band gaps for pure CCN and BOI are 2.42 and 3.13 eV, as seen in Figure 3b, which is consistent with previous reports [58,59]. Furthermore, Mott–Schottky (M–S) analysis was applied to measure the actual band edge positions of pure CCN and BOI (Figure 3c). The flat band potentials of CCN and BOI were estimated by extrapolating to C^−2^ = 0 at 800, 1000, and 1200 Hz, which are −0.51 and −0.40 V, respectively (vs. Ag/AgCl). Furthermore, the conduction band (CB) potential of n-type semiconductors is approximately equivalent to the flat-band potential [60]. Consequently, the CB potentials of CCN and BOI are approximately −0.51 and −0.40 eV, respectively, while the VB positions are 1.91 and 2.73 eV (*E_CB_* = *E_VB_ − E_g_*), respectively. The corresponding band structures of CCN and BOI are exhibited in Figure 3d.

**Figure 4:**
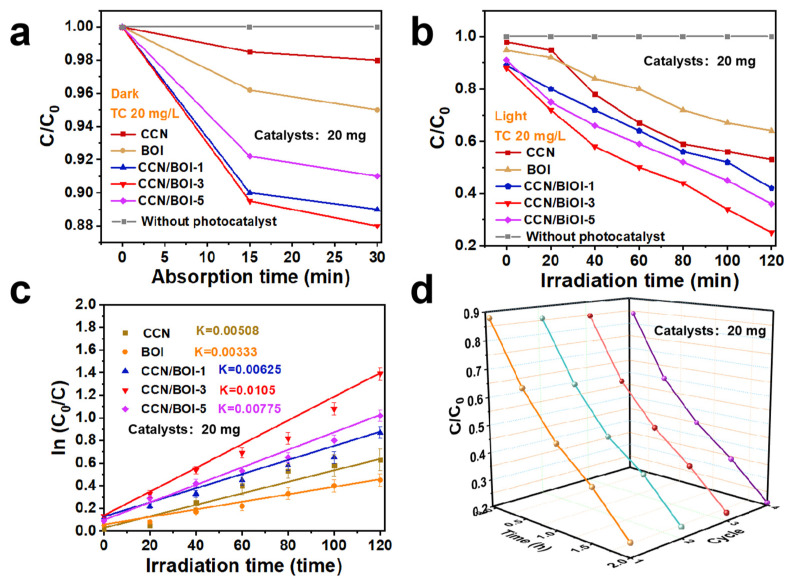
(**a**) Photodegradation of TC over different catalysts on (**a**) dark adsorption and (**b**) light irradiation. (**c**) The corresponding pseudo-first-order reaction kinetics diagram of as-prepared samples. (**d**) Photocatalytic degradation cycling runs experiment of CCN/BOI-3 sample.

The photocatalytic performance of the as-synthesized photocatalysts was evaluated by the degradation of tetracycline (TC) under visible light conditions. As displayed in Figure 4a, the TC was hardly decomposed under dark conditions, while the photocatalysts clearly degraded the TC under visible light irradiation (Figure 4b). It was observed that the photocatalytic activity of the pure 2D layered CCN and 2D BOI nanosheets was poor, with a degradation rate of 36% and 41%, respectively, which were attributed to the poor visible light absorption of both materials and the rapid recombination of photogenerated charge carriers. Significantly, the CCN/BOI-3 composite exhibited the best photocatalytic activity with the degradation rate of 79.8%, which is superior to that of previously reported CN- or BOI-based reaction systems (Table S1). Moreover, the time course variation curve of TC can be further described by the kinetic equation (Equation (1))

*ln*(*C*_0_/*C*) = *kt* (1)

It can be observed that the CCN/BOI-3 composite exhibited the highest apparent reaction rate constant *k* values (0.0105 min^−1^), which are higher than those of pure BOI (0.00333 min^−1^) and CCN (0.00508 min^−1^), respectively (Figure 4c). Simultaneously, the photocatalytic degradation activity of CCN/BOI-3 has not been changed significantly after four cycles of reaction (Figure 4d), which indicates that the CCN/BOI composite photocatalyst possesses favorable photocatalytic recycling stability. Furthermore, CCN/BOI-3 materials were employed to degrade other antibiotics such as oxytetracycline (OTC), streptomycin (STR), chlortetracycline (CTC), ciprofloxacin (CIP), and amoxicillin (AMX) under the same conditions (Figure S7), which demonstrated the universality of CCN/BOI-3 for the degradation of antibiotic contaminants.

**Figure 5:**
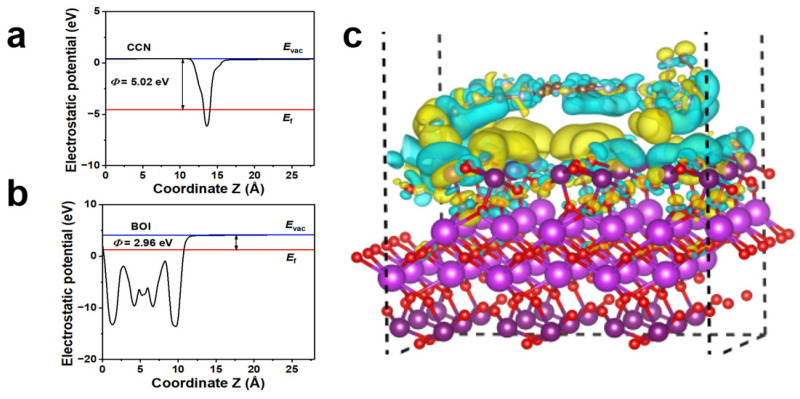
Work function profiles of (**a**) BOI and (**b**) CCN. (**c**) The charge density difference of CCN/BOI heterojunction.

To investigate the charge transfer at the atomic level, the electrostatic potentials of the CCN and BOI surfaces were evaluated based on density functional theory (DFT). From Figure 5a,b, the work function (Ф) was defined as the difference between the vacuum level (E_vac_) and the Fermi level (E_f_), which were calculated to be 5.02 eV and 2.96 eV for CCN and BOI, respectively. When the CCN is in contact with the BOI, electrons are transferred from the BOI to the CCN until the E_f_ of both compounds reaches the same level. In addition, the charge density difference (CDD) of the CCN/BOI composite was tested to further analyze the charge transfer mechanism (Figure 5c). It is observed that the interface near the CCN is dominated by yellow regions, which indicates that the BOI loses electrons, while the CCN gains electrons when the CCN/BOI heterojunction is formed. In addition, CCN does not bind to BOI at the interface, indicating that the CCN/BOI heterojunction is a VDW heterojunction [61]. The CCN/BOI VDW heterojunction promotes large interactions at the interface, which is advantageous for achieving fast charge transfer to boost photocatalytic activity. Bard charge analysis confirmed that the electron transfer of approximately 0.3 eV from BOI to CCN at the interface promoted the formation of an intrinsic electric field (IEF), driving the involvement of photogenerated electrons in the photocatalytic degradation process.

**Figure 6:**
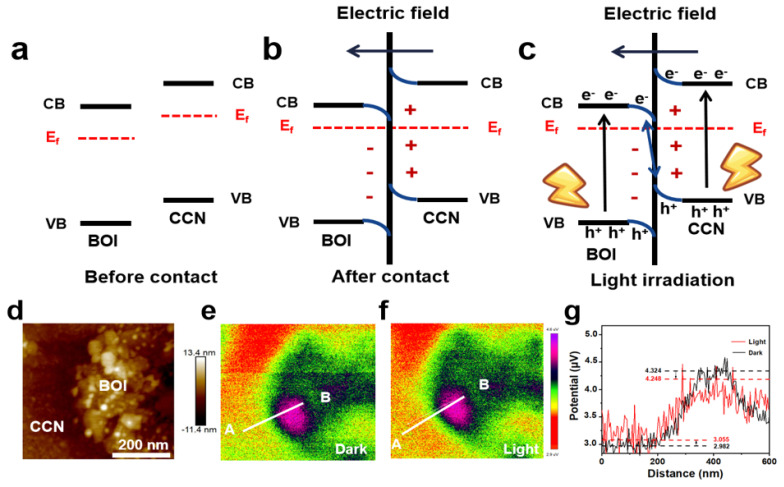
(**a**) Band configuration of CCN and BOI. (**b**) Establishment IEF of CCN/BOI heterojunction interface. (**c**) Photoexcited charge transfer of CCN/BOI S-scheme heterojunction. (**d**) AFM image of CCN/BOI. KPFM potential images of CCN/BOI (**e**) in the dark and (**f**) under light irradiation and (**g**) the corresponding surface potential curves (correspond to the line from point A to B).

Before elucidating the degradation mechanism of the TC contaminants, the redox capacity and charge transfer pathways of the as-prepared CCN/BOI photocatalyst should be investigated. The energy band structures of CCN and BOI are given in Figure 6a, derived from the UV-vis DRS, work functions, and Mott–Schottky curves. Because the work function of CCN is higher than that of BOI, when the layered BOI is connected to the CCN nanosheet to form a VDW heterojunction, the electrons of BOI are transferred to the layered CCN with higher E_f_. As a result, the CCN interface becomes negatively charged by accepting electrons, while the layered BOI interface becomes positively charged by consuming electrons [62]. Furthermore, an IEF pointing from the laminar BOI to the lamellar CCN is established (Figure 6b). Because both CCN and BOI are UV-vis-responsive semiconductors, electrons could move two parts from VB to CBs in the CCN/BOI heterojunction under the irradiation (Figure 6c). Subsequently, the IEF drives the photoexcited electrons on the CB of the weakly reducing CCN to combine with the photoexcited holes on the VB of the weakly oxidizing BOI. In this way, the strong photoexcited holes and electrons of the CCN/BOI composite are retained, leading to the formation of an S-scheme heterojunction [63]. Moreover, the charge transfer at the heterojunction interface was visualized with Kelvin Probe Force Microscopy (KPFM) measurements (Figure 6d–g). It was observed that the potential of the CCN was 1.34 μV higher than that of the BOI in the absence of light. In contrast, the surface potential of the CCN decreased and that of the BOI increased after 5 min of irradiation, which indicates that the electrons were clustered on the CCN and holes on the BOI at the surface of the heterojunction. The experimental results demonstrate the IEF generation and confirm the charge separation path of the S-scheme heterojunction.

**Figure 7:**
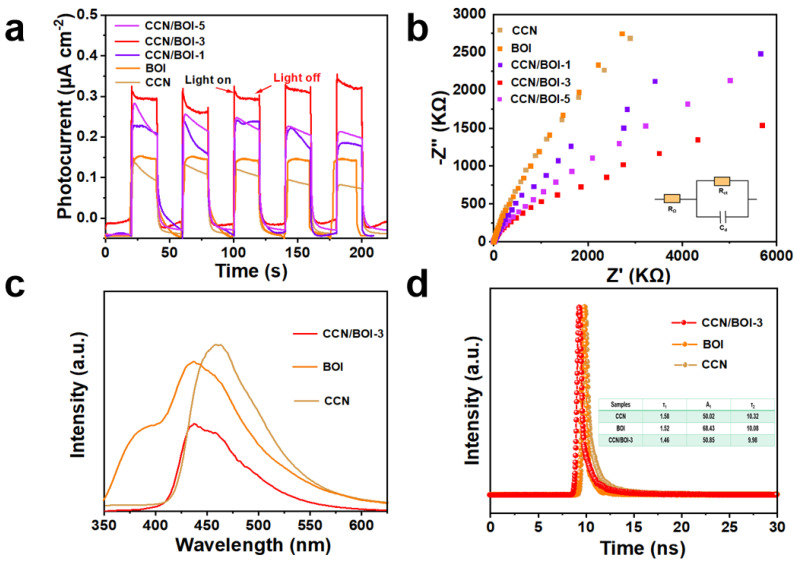
(**a**) Transient time-photocurrent curves and (**b**) EIS plots of as-prepared samples. (**c**) PL and (**d**) time-resolved PL spectra of CCN, BOI, and CCN/BOI heterojunction.

To investigate the charge separation capability of the as-synthesized photocatalysts, photocurrent response curves and electrochemical impedance spectroscopy (EIS) tests were performed [64]. As given in Figure 7a, the photocurrent density is in the order of CCN/BOI-3 > CCN/BOI-5 > CCN/BOI-1 > BOI > CCN. Apparently, the photocurrent intensity of the CCN/BOI-3 sample is higher than that of the CCN and BOI, indicating that the S-scheme VDW heterojunction can accelerate the separation and migration efficiency of photogenerated electron-hole pairs. Simultaneously, the Nyquist plot reflects the impedance corresponding to the photoexcited charge transfer. In general, the strong resistance to charge transfer leads to a larger arc radius of the semicircle in the Nyquist diagram [65]. It is observed that the arc radius of the CCN/BOI composites is significantly smaller than that of the pristine CCN and BOI, which implies that the S-scheme 2D/2D VDW heterojunction can accelerate the photoinduced charge carrier transfer (Figure 7b). In order to gain insight into the mechanism of enhanced photocatalytic performance, the process of charge transfer was investigated. Initially, exciton dissociation is the main step to generate free charges [66]. Photoluminescence (PL) spectra and corresponding time-resolved PL spectra were employed to study the dynamics of exciton dissociation generated by photo-excitation in the synthesized photocatalysts. As displayed in Figure 7c, the PL intensity of the CCN/BOI-3 composite is significantly lower compared to that of the pure CCN and BOI, suggesting that the construction of S-scheme VDW heterojunctions plays an important role in suppressing the photogenerated electron-hole pair recombination. Moreover, the corresponding time-resolved PL spectra are shown in Figure 7d. The fluorescence lifetime of CCN/BOI-3 can be calculated to be 3.59 ns, which is lower than that of CCN (3.67 ns) and BOI (3.62 ns), indicating that the S-scheme VDW heterojunction could effectively enhance the dissociation of excitons.

**Figure 8:**
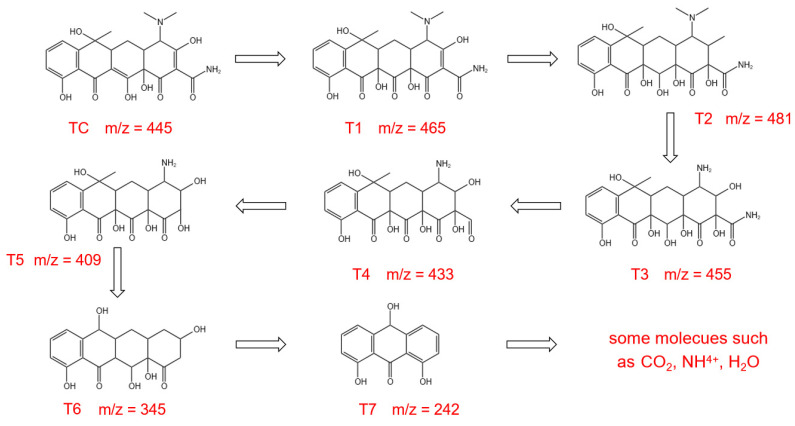
Possible photocatalytic degradation pathways of TC over CCN/BOI heterojunction.

In order to investigate the degradation pathway of TC, the degradation intermediates of TC were detected by liquid chromatography-mass spectrometry (LC-MS) analysis, and the results are displayed in Figure S4. An intense anionic absorption peak can be observed at *m/z* = 445, which is assigned to the deprotonated TC molecule [67]. As the reaction time increase, the peaks of the TC molecule gradually decrease and some new peaks belonging to the intermediate appeared. A series of degradation products can be identified in the MS spectra, such as *m/z* = 465, 481, 455, 433, 409, 342, and 242. Based on the detected intermediates, the possible degradation pathways of TC were analyzed and are exhibited in Figure 8. Initially, the TC molecules are oxidized by hydroxyl radicals, thus producing the intermediates T1 (*m/z* = 465). T1 is further oxidized by the hydroxyl groups to T2 (*m/z* = 481). By loss of the N-methyl group, product T2 is further converted to compound T3 (*m/z* = 455), with subsequent loss of amino leading to the formation of compounds T4 (*m/z* = 433). T5 (*m/z* = 409) is formed by T4 removing the c functional group [5]. The gradual separation of the branched chain groups of T5 forms T6 (*m/z* = 345), and after continuous redox reaction, opens the six-element ring structure to form T7 (*m/z* = 242). Finally, with the extension of the irradiation time, all intermediates are further degraded to H_2_O, NH^4+^, and CO_2_, etc. [68].

**Figure 9:**
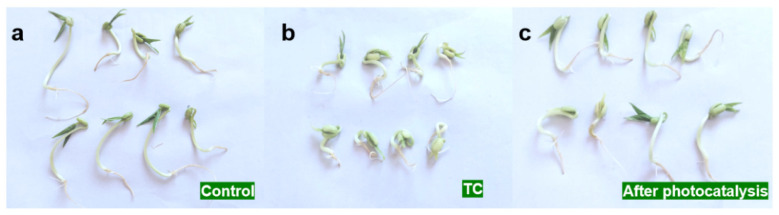
Growth of mung beans in different solutions: (**a**) control; (**b**) TC solution; and (**c**) TC solution after photocatalysis.

The biotoxicity of the TC solution after photocatalysis has attracted much attention due to its negative effects on the daily environment. Mung bean sprouts were cultured in different solution systems to explore the changes of TC biotoxicity before and after photocatalytic degradation. First, mung bean sprout seeds were planted in petri dishes containing deionized water, TC solution, and photocatalyst-treated TC solution, respectively, and the pictures after seven days of planting are presented in Figure 9. From Figure 9a, it can be observed that all bean sprouts grown in deionized water germinated successfully and grew well. In contrast, the mung beans grown in untreated TC solution germinated successfully but grew poorly (Figure 9b). Surprisingly, all mung beans in the treated TC solution germinated as it was in an aqueous solution (Figure 9c), indicating that the photocatalyst degraded most of the biotoxic molecules, and the biotoxicity of the treated TC solution was significantly reduced. In addition, mung bean sprouts grown in the photocatalyst-treated TC solution were slightly worse than those grown in deionized water, which was mainly due to a small fraction of residual TC toxicity in the water [69].

**Figure 10:**
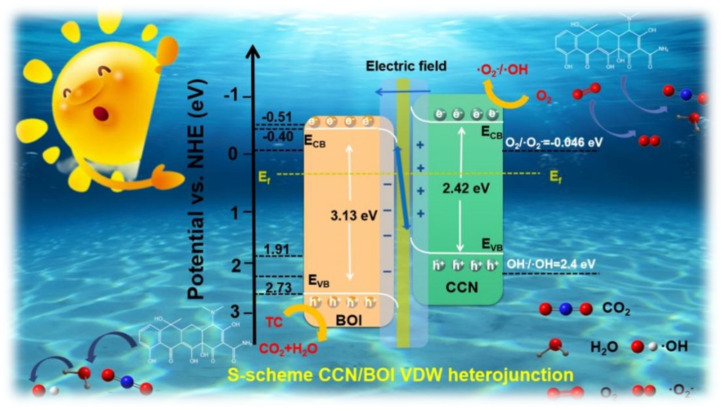
Proposed photocatalytic reaction mechanism for the degradation of TC over CCN/BOI heterojunction.

Based on the above experimental results and theoretical calculation analysis, the possible photocatalytic TC degradation mechanism of S-scheme CCN/BOI VDW heterojunctions is presented in Figure 10. DFT calculations indicate that the Fermi level of CCN is higher than that of BOI. Consequently, upon material contact, electrons tend to spontaneously migrate from BOI to CCN until the Fermi level reaches equilibrium. The charge rearrangement process leads to band-edge bending, creating an established IEF at the interface between the CCN and the BOI. Under visible light and irradiation, the CCN and BOI materials are excited to generate electrons and holes. With the built-in electric field, electrons in BOI CB readily recombine with holes on CCN VB, leading to electron transfer along the path of the S-scheme. Furthermore, the strong interaction between the 2D CCN and BOI nanosheets can promote charge transfer and facilitate the rearrangement of energy level, which has been demonstrated by the M–S diagram. As displayed in Figure S5, the CCN/BOI material exhibits a lower positive slope of the curve than that of CCN and BOI, indicating an enhanced concentration of charge carriers in the VDW heterostructure [70]. Furthermore, the holes on the VB of BOI could trap free OH^−^ in water and further oxidize it to hydroxy radicals (OH) [71]. Meanwhile, the electrons accumulated on the CB of CCN can continuously reduce O_2_ to superoxide radical (O_2_^−^) [72]. Moreover, distinctive peaks for DMPO-·OH and DMPO-·O_2_^−^ can be detected during the photocatalytic reaction via electron spin resonance (ESR) measurements, confirming the presence of ·OH and ·O_2_^−^ active species (Figure S6). Ultimately, the major active substances are able to oxidatively reduce TC to H_2_O, CO_2_, etc. The reaction mechanism of CCN/BOI photocatalytic degradation of TC can be summarized as follows:

CCN/BOI + *hν* → *e^−^* (CCN/BOI) + *h^+^* (CCN/BOI) (2)

*e^−^* (BOI) + *h^+^* (CCN) → (BOI) *h^+^* + (CCN) *e^−^* (3)

*e^−^* (CCN) + O_2_ → CCN + ·O_2_^−^ (4)

*h^+^* (BOI) + OH^−^ → BOI + ·OH (5)

·O_2_^−^ + TC → products (6)

*h^+^* + TC → products (7)

·OH + TC → products(8)

## 3. Materials and Methods

### 3.1. Chemical Reagents

Melamine (C_3_N_3_(NH_2_)_3_), bismuth nitrate pentahydrate (Bi(NO_3_)_3_**⋅**5H_2_O), nitric acid (HNO_3_), and potassium iodate (KIO_3_) were all analytical-grade reagents and purchased from Sinopharm Reagent Co., Ltd (Shanghai, China). The tetracycline used for the experiment was purchased from Aladdin Reagent Co., Ltd (Shanghai, China).

### 3.2. Synthesis of Crystalline CN (CCN) Nanosheets 

Crystalline carbon nitride (CCN) was prepared based on a previously reported one-step rapid polymerization strategy [73]. A certain amount of melamine (3 g) was placed in a crucible and calcined directly at 550 °C in a muffle furnace for 4 h. Finally, the temperature was reduced to room temperature (the cooling rate was 10 °C/min) and yellow powder was obtained by grinding as CCN. For comparison, the bulk g-C_3_N_4_ was fabricated by calcinating 10 g melamine at 500 °C in a muffle furnace for 4 h (heating rate: 10 °C/min) with a covered crucible. After cooling down, the collected yellow sample was ground to powder and denoted as BCN.

### 3.3. Synthesis of BiOIO_3_ (BOI) Nanosheets 

The flake BiOIO_3_ was prepared by a hydrothermal procedure. A quantity of Bi(NO_3_)_3_**⋅**5H_2_O (1.0 g) and KIO_3_ (0.4 g) were dissolved in 60 mL of pure water under continuous stirring for 30 min, with the addition of 2 mL HNO_3_ solution. Subsequently, the mixed suspension was transferred to a 100-mL Teflon-lined autoclave and heated at 150 °C for 15 h. The obtained BiOIO_3_ product was then washed three times with pure water and ethanol, before drying in an oven at 60 °C for 8 h and being denoted as BOI.

### 3.4. Synthesis of CCN/BOI Heterojuntions

For the synthesis of the CCN/BOI composites, 1.0 g Bi(NO_3_)_3_**⋅**5H_2_O and 2 mL HNO_3_ were added to 60 mL of pure water then stirred for 15 min. Subsequently, a quantity of CCN and 0.4 g KIO_3_ were added to the above mixture under continuous magnetic stirring for 30 min. The suspension was then transferred to a 100-mL Teflon-lined autoclave and heated at 150 °C for 15 h. After cooling to room temperature, the product was centrifuged and washed several times with water and ethanol, respectively. Finally, the processed product was dried in an oven at 60 °C for 8 h. For comparison, the CCN/BOI preparation was supplemented with 1, 3, and 5 wt.% of CCN content. For convenience, the resulting complexes are recorded as CCN-BOI-1, CCN-BOI-3, and CCN-BOI-5, respectively. 

## 4. Conclusions

In summary, the 2D/2D S-scheme CCN/BOI van der Waals heterojunction was successfully fabricated via a simple hydrothermal method. The 2D/2D structured CCN/BOI allows the synthesized photocatalysts to possess a large interface area, providing a channel for charge transfer, while the van der Waals force between the components accelerates carrier separation and transfer. Furthermore, the S-scheme heterojunctions enhance the redox ability of 2D/2D CCN/BOI composites, contributing a significant driving force for subsequent photocatalytic degradation of TC. As expected, the as-synthesized optimal CCN/BOI-3 photocatalyst displayed an excellent degradation performance and an outstanding TC degradation rate within 120 min, which is 2 times and 2.7 times higher than that of pure CCN and BOI, respectively. This work provides guidance for the design of efficient photocatalysts with 2D/2D S-scheme van der Waals heterojunctions.

## Data Availability

Not applicable.

## Supplementary Materials

The following supporting information can be downloaded at: https://www.mdpi.com/article/10.3390/molecules28135098/s1, Figure S1 (a) SEM image of CCN/BOI-3 and (b) EDS spectrum of CCN/BOI-3. Figure S2 XRD patterns of BCN and CCN. Figure S3 Digital photographs of (a) CCN, (b) BOI, (c) CCN/BOI-1, (d) CCN/BOI-3, (e) CCN/BOI-5. Figure S4 LC-MS spectra of possible intermediates for the degradation of TC over CCN/BOI heterojunction. Figure S5 M-S plots of CCN, BOI and CCN/BOI-3. Figure S6 ESR spectra of the (a) DMPO-•OH and (b) DMPO-•O_2_^–^ over CCN/BOI in the dark and under visible light irradiation. Figure S7 Degradation rates of CCN/BOI-3 photocatalysts for different kinds of antibiotics: tetracycline (TC), oxytetracycline (OTC), streptomycin (STR), chlortetracycline (CTC), ciprofloxacin (CIP) and amoxicillin (AMX) at concentrations of 20 mg/L. Table S1 Comparison of CCN/BOI reaction systems photocatalytic performance with other previously reported reaction systems [74,75,76,77,78,79].

## Sample Availability

Samples of the compounds are available or not available from the authors.
