# Supplementary material for: Construction of S-Scheme 2D/2D Crystalline Carbon Nitride/BiOIO3 van der Waals Heterojunction for Boosted Photocatalytic Degradation of Antibiotics
Source: Molecules. 2023 Jun 29;28(13):5098. doi: 10.3390/molecules28135098 (PMC10343194; doi:10.3390/molecules28135098)
Supplement: Supplementary file 1 [file molecules-28-05098-s001.zip › molecules-2473482-supplementary.pdf]

Supporting Information

Construction of S-Scheme 2D/2D Crystalline Carbon Nitride/$BiOIO_3$ van der Waals Heterojunction for Boosted Photocatalytic Degradation of Antibiotics

Xiangyuan Kong [1,*], Longwen Cao [2], Yuxing Shi [2], Zhouze Chen [2], Weilong Shi [2,*] and Xin Du [3,*]

1 School of Physical Science and Engineering, Beijing Jiaotong University, Beijing 100091, China

2 School of Material Science and Engineering, Jiangsu University of Science and Technology, Zhenjiang 212003, China; czz990707@163.com (Z.C.)

3 College of Chemistry, Zhengzhou University, Zhengzhou 450001, China

* Correspondence: 20272038@bjtu.edu.cn (X.K.); shiwl@just.edu.cn (W.S.); dux@zzu.edu.cn (X.D.)

1. Characterizations

Powder X-ray diffraction (XRD) patterns were obtained from a German Bruker-AXSSM D8 with Cu Kα radiation at a scanning speed (2θ) of 10° min^{-1} from 10° to 80°. X-ray photoelectron spectroscopy (XPS) was employed by a Krato Axis Ultra DLD to study the chemical nature of C, N, and S with Al Kα source. Room temperature UV-vis diffuse-reflectance spectra (UV–vis DRS) were collected using UV-2450 spectrometer to research optical absorption properties of samples. Photoluminescence (PL) spectroscopy was carried out on a Perkin-Elmer LS 55 luminescence spectrometer. The Fourier transform infrared spectroscopy (FT-IR) spectra of samples were obtained on a FT-IR650 spectrometer. The scanning electron microscopy (SEM) images were gained on a Philips FEI Quanta 200 FEG to study the morphologies of samples. Transmission electron microscope (TEM) and high-resolution transmission electron microscope (HRTEM) images were carried out on FEI-Tecnai F20. Time-resolved photoluminescence (TRPL) spectra were monitored by Edinburgh FLS980 transient fluorescence spectrophotometer. The intermediate products were analyzed by liquid chromatography-mass spectrometry (LC-MS) (Ultimate 3000 UHPLC-Q Exactive). The electron spin resonance (ESR) of free radicals was measured with a Bruker A300

spectrometer under visible light irradiation (λ > 420 nm).

2. Photoelectrochemical measurements

The photoelectrochemical tests of samples were obtained by using a CHI 660E electrochemical workstation with a three-electrode system. The measurement process of the Mott-Schottky (M-S) curves, photocurrent response and the electrochemical impedance spectra (EIS) was as follows: 0.5 M Na_2SO_4 solution was employed as electrolyte, Pt wire, calomel electrode and the prepared samples were used as the counter electrode, the reference electrode and the working electrode, respectively. Besides, 300 W Xe lamp with filter of λ > 420 nm was used as the visible light source. Then, 5 mg sample was dissolved in the mixed solution of 30 μL naphthol and 1 mL deionized water under continuous ultrasonic conditions for 10 h. Subsequently, the prepared samples were dropped on the surface of the cleaned FTO glasses and naturally dry to gain the electrodes for tests.

3.Photocatalytic degradation experiments

The photocatalytic degradation experiments were carried out in a parallel photocatalytic reactor (CEL-LAB200E7, Beijing Zhongjiao Jinyuan Technology Co., Ltd) using a 30W LED as the visible light source (λ: 410-760 nm) under ambient conditions. The detailed experimental procedure was as follows: 20 mg of catalyst was dispersed in a configured 50 mL tetracycline (TC) solution (20 mg/L) and the dark reaction was carried out for 30 min protected from light. After reaching adsorption equilibrium, the light reaction is carried out by switching on the LED lamp for 2 hours and the supernatant (2 mL) is centrifuged at 20 min intervals. Subsequently, the solution obtained by centrifugation was passed through a UV-vis spectrophotometer to measure the concentration of the remaining TC.

4. Computational Methods (DFT)

The first-principles Density Functional Theory (DFT) was calculated using the Vienna Simulation Package (VASP) on the website (https://www.materialsproject.org/materials/mp-990448/#). The projector augmented wave (PAW) potential is used to represent the calculation of the hybrid function proposed by the PBE interaction, and the Kohn-Sham one-electron valence state is extended based on a plane wave with a cut-off energy of 400 eV.

5. Biological toxicity test on mung growth

Three plates were placed in an incubator with a constant shading temperature (25 °C), and 8 mung beans were added to each dish. Different solution, including deionized water (10 mL), diluted TC solution (9 mL deionized water and l mL TC solution with concentration of 20 mg/L) and diluted TC solution treated after photocatalytic degradation (9 mL deionized water and 1 mL TC solution of 20 mg/L after degradation) were selected as nutrient solution to cultivate bean sprouts. The experiment lasted 7 days and renewed the nutrient solution every 24 h. Finally, mung bean sprouts can be harvested after 7 days.

6. Figures and tables

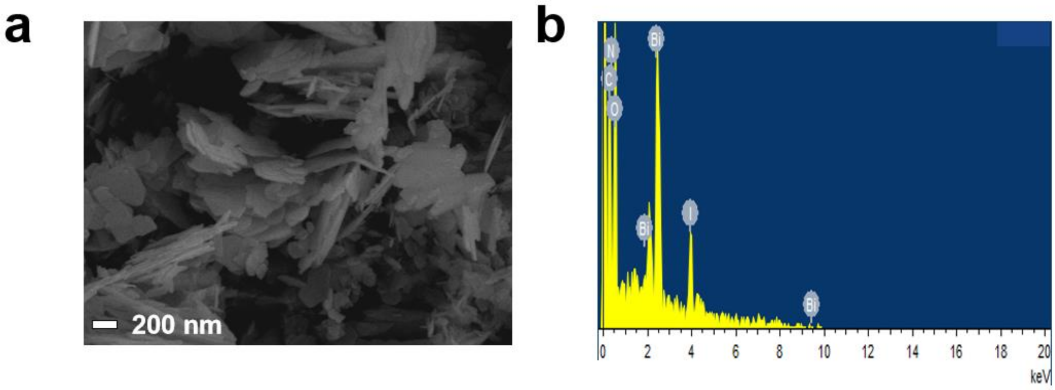

Figure S1 (a) SEM image of CCN/BOI-3 and (b) EDS spectrum of CCN/BOI-3.

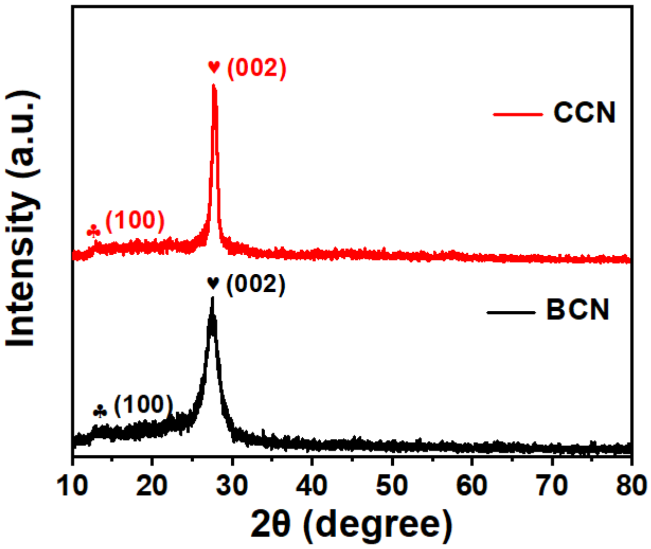

Figure S2 XRD patterns of BCN and CCN.

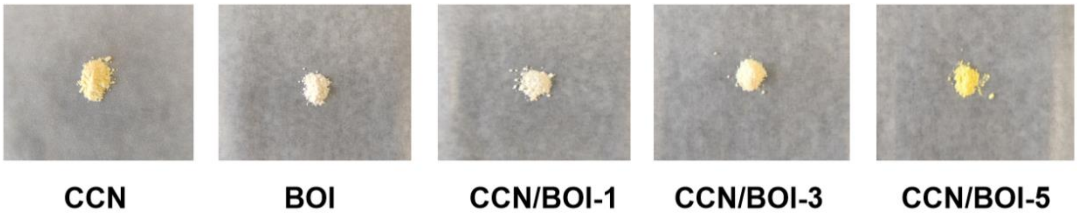

Figure S3 Digital photographs of (a) CCN, (b) BOI, (c) CCN/BOI-1, (d) CCN/BOI-3, (e) CCN/BOI-5.

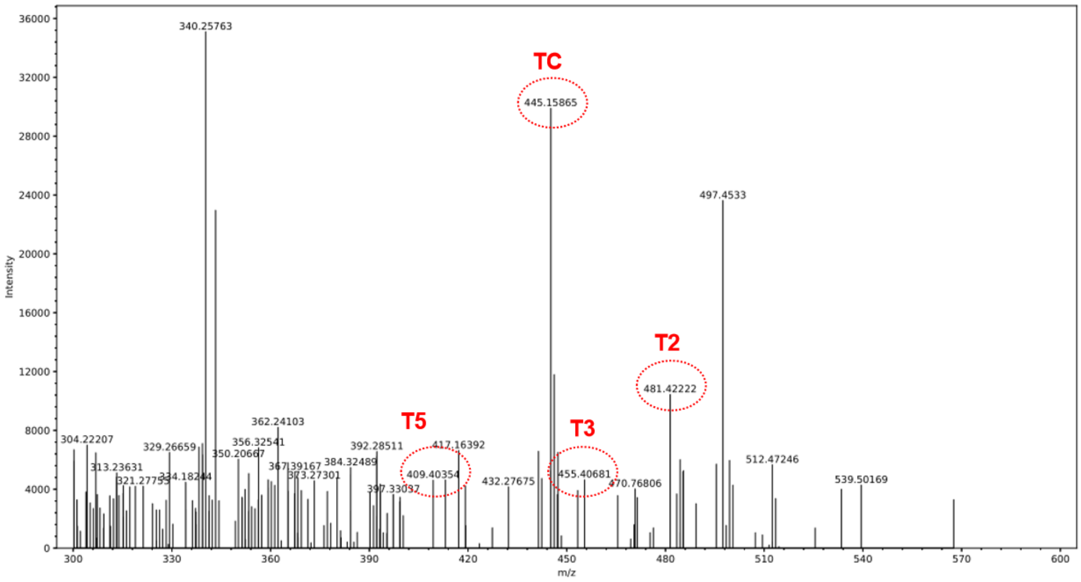

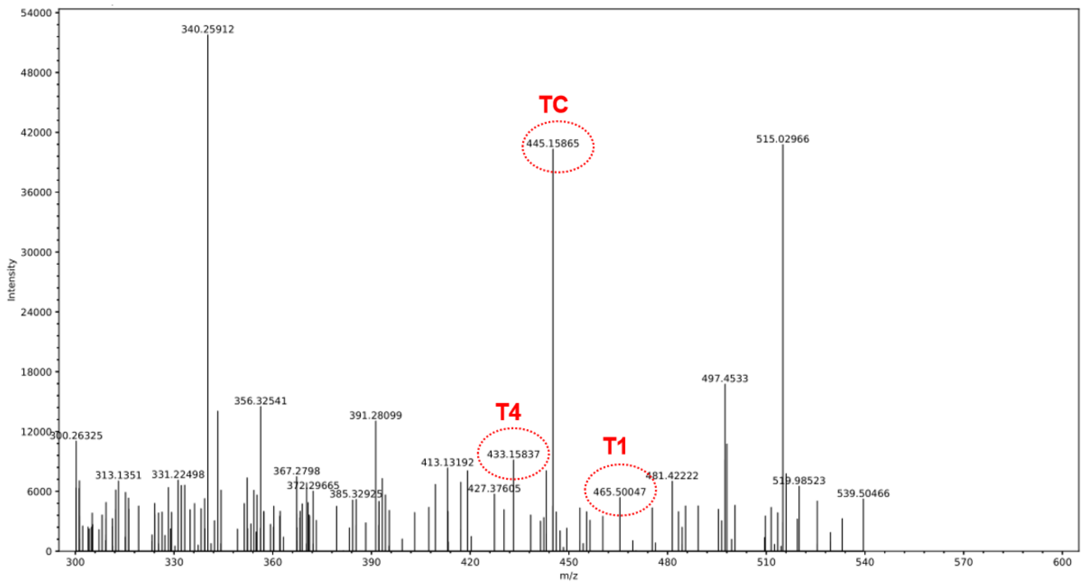

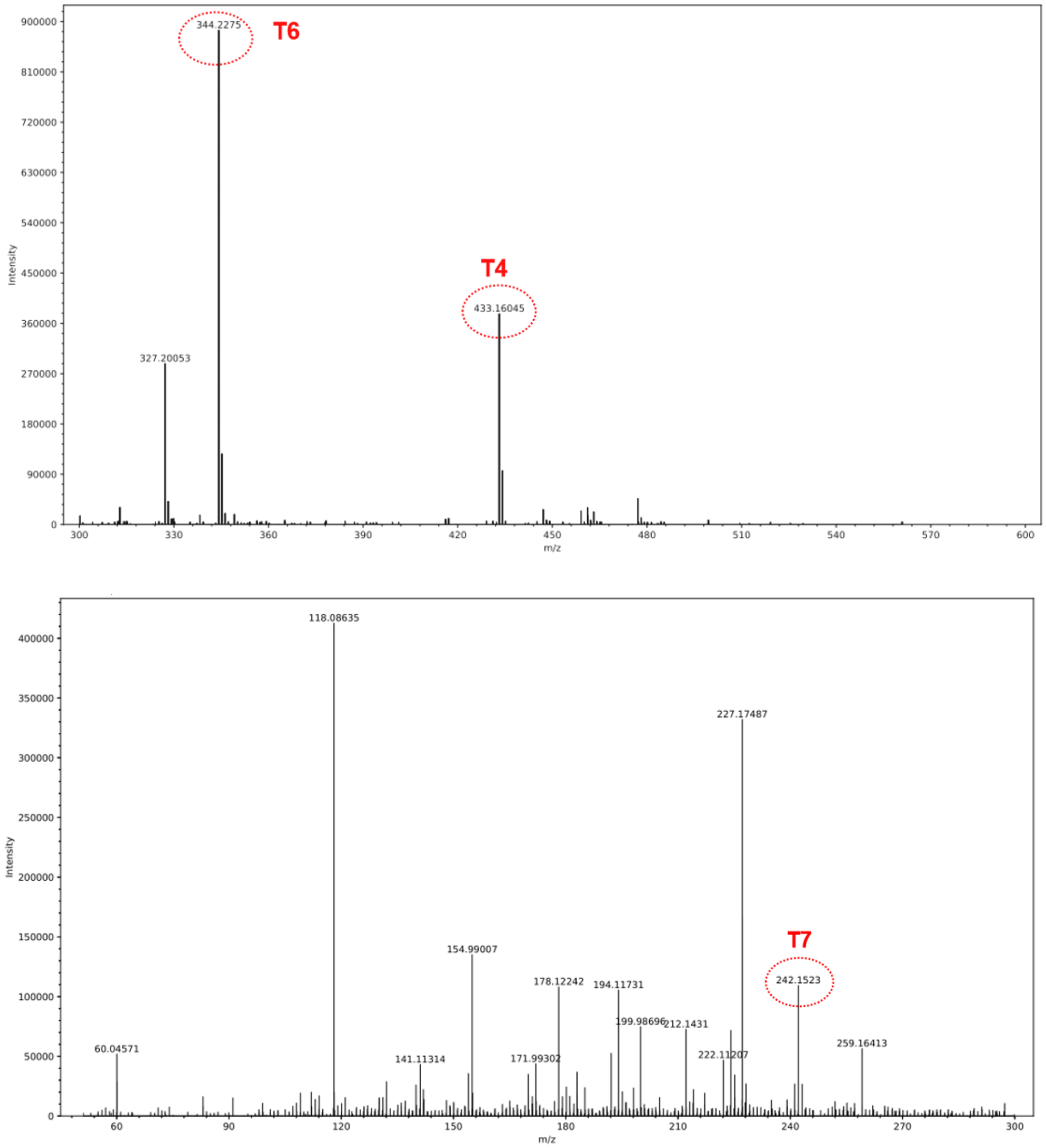

Figure S4 LC-MS spectra of possible intermediates for the degradation of TC over CCN/BOI heterojunction.

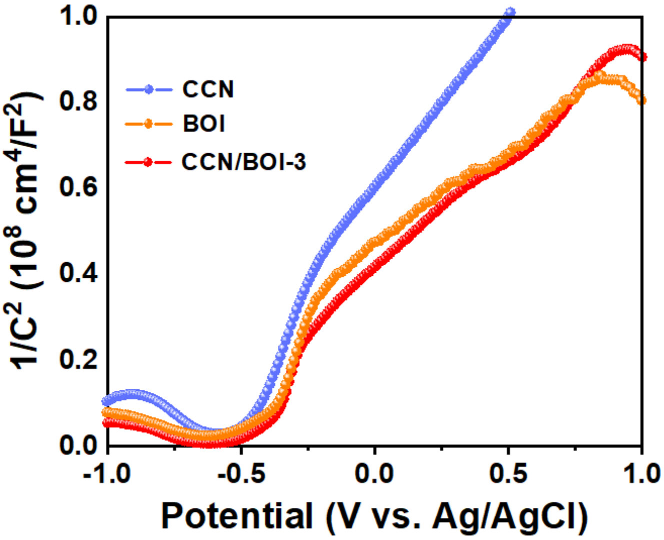

Figure S5 M-S plots of CCN, BOI and CCN/BOI-3.

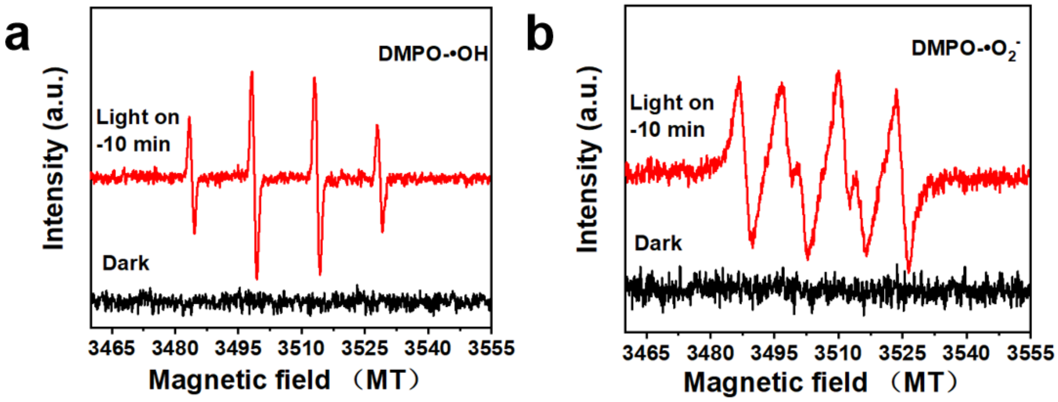

Figure S6. ESR spectra of the (a) DMPO-•OH and (b) DMPO-•O_2^- over CCN/BOI in the dark and under visible light irradiation.

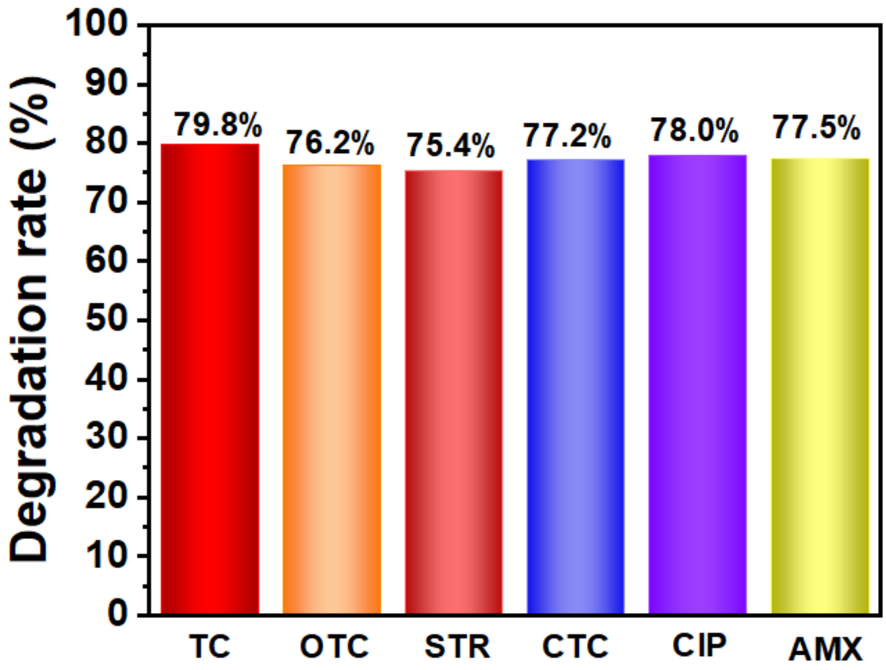

Figure S7 Degradation rates of CCN/BOI-3 photocatalysts for different kinds of antibiotics: tetracycline (TC), oxytetracycline (OTC), streptomycin (STR), chlortetracycline (CTC), ciprofloxacin (CIP) and amoxicillin (AMX) at concentrations of 20 mg/L.

Table S1 Comparison of CCN/BOI reaction systems photocatalytic performance with other previously reported reaction systems.

Samples	Pollutions	$C_{catalyst}$ (g/L)	$C_{pollutions}$ (mgL^{-1})	DR %	Rate constant min^{-1}	References
PTI/g-C_3N_4	TC-HCl	1.0	10	68 (150 min)	0.007	[74]
Ag/g-C_3N_4	TC	0.2	30	77 (120 min)	-	[75]
CNU-SA-2	TC	0.5	20	82.3 (180 min)	0.0095	[76]
0.01TCN	TC	0.5	25	83.5 (90 min)	0.0241	[77]
CQDs/$BiOIO_3$	TC	0.025	-	70 (120 min)	0.000937	[78]
$BiOIO_3$/BiOBr	TC	1.0	20	74.9 (80 min)	-	[79]
CCN/BOI-3	TC	0.25	20	79.8 (120 min)	0.0105	This work

References

74. Dong, X.Y.; Huang, X.Q.; Wang, D.B.; Lei, Y.; Han, J.C.; Liang, X.F.; Wei, Q.L. Constructing crystalline needle-mushroom-like/ amorphous nanosheet carbon nitride homojunction by molten salt method for photocatalytic degradation of tetracycline hydrochloride, J. Mater Sci-Mater El. **2022**, 33, 6043-6058.
75. Zhang, P.; Wang, J.Q.; Gong, J.; Wang, K.; Li, Y.; Wu, X.Y. Fabrication of Ag/carbon nitride photocatalysts and their enhanced photocatalytic performance for tetracycline degradation, Funct. Mater. Lett. 2020, *13*, 2051033.
76. Chen, M.M.; Li, M.X.; Li, P.; Lee, S.L.J.; Tang, J.J.; Li, Q.; Lin, S.J. Enhanced visible light-driven photodegradation of tetracycline by salicylic acid-modified graphitic carbon nitride and toxicity assessment, Environ. Sci. Pollut. R. **2022**, *29*, 90768–90778.
77. Wang, B.; Cao, Q.T.; Li, G.M.; Zhang, J. Preparation of non-polluting Tb-doped mesoporous carbon nitride photocatalyst and study on the efficacy and mechanism of degradation of antibiotics in water, Environ. Sci. Pollut. R. **2022**, *29*, 36337–36350.
78. Wang, X.; Jing, H.X.; Gao, Y.L.; Xin, Y.Z.; Li, Q.L. CTAB assisted hydrothermal synthesis of 0D / 2D structure carbon quantum dots $BiOIO_3$ composite for photocatalytic degradation of tetracycline, Inorg. Chem. Commun. **2022**, *146*, 110209.
79. Kuang, X.; Fu, M.; Kang, H.; Lu, P.; Bai, J.W.; Yang, Y.; Gao, S.X. A $BiOIO_3$/BiOBr n-n heterojunction was constructed to enhance the photocatalytic degradation of TC, Opt. Mater. **2023**, *138*, 113690.
